# Discovery of three novel neutralizing antibody epitopes on the human astrovirus capsid spike and mechanistic insights into virus neutralization

**DOI:** 10.1128/jvi.01619-24

**Published:** 2025-01-23

**Authors:** Sarah Lanning, Nayeli Aguilar-Hernández, Vitor Hugo B. Serrão, Tomás López, Sara M. O'Rourke, Adam Lentz, Lena Ricemeyer, Rafaela Espinosa, Susana López, Carlos F. Arias, Rebecca M. DuBois

**Affiliations:** 1 Department of Molecular Cell and Developmental Biology, University of California Santa Cruz 214546 , Santa Cruz, California, USA; 2 Department of Biomolecular Engineering, University of California Santa Cruz 8787 , Santa Cruz, California, USA; 3 Departamento de Genética del Desarrollo y Fisiología Molecular, Instituto de Biotecnología, Universidad Nacional Autónoma de México 7180 , Mexico City, Mexico; 4 Biomolecular Cryo-electron Microscopy Facility, University of California Santa Cruz 8787 , Santa Cruz, California, USA; 5 Department of Chemistry and Biochemistry, University of California Santa Cruz 8787 , Santa Cruz, California, USA; University of Michigan Medical School, Ann Arbor, Michigan, USA

**Keywords:** human astrovirus, capsid, surface antigens, monoclonal antibodies, protein structure-function, X-ray crystallography, cryo-EM, neutralization

## Abstract

Human astroviruses (HAstVs) are a leading cause of viral childhood diarrhea that infects nearly every individual during their lifetime. Although human astroviruses are highly prevalent, no approved vaccine currently exists. Antibody responses appear to play an important role in protection from HAstV infection; however, knowledge about the neutralizing epitope landscape is lacking, as only three neutralizing antibody epitopes have previously been determined. Here, we structurally define the epitopes of three uncharacterized HAstV-neutralizing monoclonal antibodies: antibody 4B6 with X-ray crystallography to 2.67 Å, and antibodies 3H4 and 3B4 simultaneously with single-particle cryogenic-electron microscopy to 3.33 Å. We assess the epitope locations relative to conserved regions on the capsid spike and find that while antibodies 4B6 and 3B4 target the upper variable loop regions of the HAstV spike protein, antibody 3H4 targets a novel region near the base of the spike that is more conserved. Additionally, we found that all three antibodies bind with high affinity, and they compete with receptor FcRn binding to the capsid spike. These studies inform which regions of the HAstV capsid can be targeted by monoclonal antibody therapies and could aid in rational vaccine design.

**IMPORTANCE:**

Human astroviruses (HAstVs) infect nearly every child in the world, causing diarrhea, vomiting, and fever. Despite the prevalence of human astroviruses, little is known about how antibodies block virus infection. Here, we determined high-resolution structures of the astrovirus capsid protein in a complex with three virus-neutralizing antibodies. The antibodies bind distinct sites on the capsid spike domain. The antibodies block virus attachment to human cells and prevent capsid spike interaction with the human neonatal Fc receptor. These findings support the use of the human astrovirus capsid spike as an antigen in a vaccine to prevent astrovirus disease.

## INTRODUCTION

Human astroviruses (HAstVs) are a significant cause of childhood viral diarrhea worldwide, with over 35% of children having had a HAstV infection by age 2 (1). These small nonenveloped RNA viruses are typically spread by fecal-oral or salivary routes (2, 3). While HAstV infections are typically self-limiting in immunocompetent people, they can persist as a chronic infection in immunocompromised individuals (4–6). Young children and immunocompromised individuals are the populations most at risk for HAstV disease, particularly in lower-income or tropical countries where higher burdens of diarrheal disease and additional comorbidities may exist (7, 8). The classical HAstV clade includes eight serotypes (HAstV1-8), with serotype 1 being the most prevalent worldwide (9–11). Divergent VA and MLB clades, which may have arisen from animal astroviruses, have been found to cause fatal encephalitis in immunocompromised individuals, and additionally, there has been a report of central nervous system involvement by classical HAstV (12, 13). Encephalitic symptoms can also be caused by some animal astroviruses, such as mink and bovine astroviruses (14, 15). Notably, astrovirus-associated encephalitis has been found to be endemic in mink and pig farms, where animals are maintained under intensive production conditions (16). Despite HAstV’s prevalence and global health impacts, there are currently no vaccines or HAstV-specific therapeutics available.

However, the development of vaccines against HAstV seems feasible, since some evidence suggests the presence of lasting HAstV immunity induced by prior infection (1). Seroprevalence to HAstV in adults is very high (>90%) (17, 18), and HAstV disease is rarer in adults than in children (19). Additional studies have supported that the presence of anti-HAstV antibodies may help to protect from severe HAstV disease (3), and one case study showed improvement in a patient with chronic HAstV disease after immunoglobulin therapy (20). However, the mechanism of how antibodies neutralize HAstV is not well understood, in part due to a lack of knowledge surrounding how antibodies interact with the viral capsid, and which parts of the exposed viral capsid are critical for its function.

The HAstV virion consists of a small proteinaceous icosahedral capsid roughly ~40 nm in diameter, which shelters a ~7 kb single-stranded positive sense polyadenylated RNA genome. The immature capsid is made up of 180 units of capsid protein originating from open reading frame 2 and displays a T = 3 symmetry. The capsid protein is initially expressed as a 90 kDa protein (VP90), which undergoes an intracellular caspase cleavage that is important for viral release from the cell, resulting in a ~70 kDa (VP70) protein after the loss of its C-terminal acidic domain (21, 22). In this state, the virus remains immature and must undergo further extracellular protease cleavage(s) to reach its mature infectious form. The exact extracellular protease used *in vivo* for this cleavage event is unknown, but *in vitro* cleavage with trypsin results in a 10^5^-fold increase in infectivity (23). This extracellular protease cleavage event cleaves VP70 into the core domain (VP34), and spike domain (VP25/VP27), and additionally removes 60 of the initial 90 dimeric spikes along fivefold symmetry axes, resulting in 30 dimeric spikes (VP27) remaining on the mature capsid along the twofold symmetry axes (24, 25). The spike domain is known to be important for attachment and entry of the virus, and antibodies that target the spike domain have been found to neutralize HAstV in cell culture, whereas antibodies that target the core domain have not been reported to neutralize HAstV (26, 27). Recently, two separate groups reported the identification of the neonatal Fc receptor (FcRn) as an important host receptor for human astrovirus entry, and FcRn was found to bind the HAstV capsid spike (28, 29). However, information about which regions of the spike are important for this interaction and how antibodies may interfere with this function remains mostly unexplored.

Only three HAstV-neutralizing antibody epitopes have been structurally defined, revealing two neutralizing antigenic sites on the HAstV spike, since two of the neutralizing antibodies (3E8, PL-2) have overlapping epitopes (30, 31). Both neutralizing epitope regions were located around a conserved putative receptor binding site on the surface of the HAstV spike, known as the “P-site,” and these antibodies were additionally shown to block spike attachment to cells (30). Whether these antibodies prevent FcRn binding or some other host factor interaction remains unknown. Furthermore, whether additional neutralizing antigenic sites exist on the HAstV spike remains unknown. Here, we structurally define three novel neutralizing antibody epitopes, assess their epitope location relative to conserved regions of the HAstV spike, and provide evidence supporting their mechanism of HAstV neutralization.

## MATERIALS AND METHODS

### Cells and viruses

Caco-2 cells, clone C2Bbe1 (ATCC), were propagated in high-glucose Dulbecco’s modified Eagle’s medium (Sigma) supplemented with nonessential amino acids (Gibco) and 15% fetal bovine serum (Cansera) in a 10% CO_2_ atmosphere at 37°C. HAstV serotypes 1 and 2 have been described previously (27). All viral strains were activated with trypsin and grown as described before (32).

### Expression and purification of recombinant HAstV1 and HAstV2 capsid spike proteins

Recombinant HAstV1 and HAstV2 spikes were produced as described previously (32, 33). Briefly, cDNA corresponding to HAstV1 capsid protein residues 429 to 645 (GenBank: AAC34717.1) or HAstV2 Oxford strain capsid protein residues 429 to 644 (GenBank: KY964327.1) were cloned into pET52B with a C-terminal thrombin cleavage site and a 10-histidine purification tag sequence. Recombinant spikes were expressed in *Escherichia coli* BL21(DE3) and purified from soluble lysates by HisTrap metal-affinity chromatography. Purified HAstV spikes were dialyzed into Tris-buffered saline (TBS: 10 mM Tris pH 8.0 and 150 mM NaCl).

### Expression and purification of recombinant monoclonal antibody Fabs 3B4, 3H4, and 4B6

The protein-coding sequence of antibodies 3H4, 3B4, and 4B6 heavy and light chains were determined as described previously (34). The protein-coding sequences of the 3H4, 3B4, and 4B6 light chain and the 3H4, 3B4, and 4B6 heavy chain antigen-binding fragment (Fab) were cloned into separate pCMV plasmids in-frame with an N-terminal human IgG1 signal sequence. The Fab heavy chains were cloned in-frame with a C-terminal thrombin-cleavable double StrepTagll affinity tag. A total of 120 µg of heavy chain plasmid and light chain plasmid combined were added to 8 × 10^7^ CHO-S cells in an OC-400 cuvette (MaxCyte) and were electroporated. CHO-S cells were resuspended in CD-OptiCHO media (Gibco: #12681029) and fed CHO feed (CHO CD EfficientFeed A [Gibco: #A1023401] supplemented with 7 mM L-glutamine, 5.5% glucose, and 23.4 g/L yeastolate) every 24 h. CHO-S cells were given a final concentration of 1 mM sodium butyrate and maintained at 32°C, 8% CO_2_, 85% humidity, 135 rpm, 24 h after electroporation for 8–10 days. CHO-S cells were centrifuged, and the resulting supernatants were given 1× protease inhibitor cocktail (Millipore 539137), BioLock (Iba Lifesciences 2-0205-050) to block free biotin in the media, and Strep Wash Buffer (50 mM Tris pH 7.4, 150 mM NaCl, 1 mM EDTA), and were 0.22 µm filtered. Samples were loaded onto a regenerated StrepTrap HP 5 mL column (Cytiva), washed with Strep Wash Buffer, and eluted with an increasing linear gradient of Strep Elution Buffer (Strep Wash Buffer + 2.5 mM desthiobiotin).

### Expression and purification of recombinant monoclonal antibody scFv 3B4, 3H4, 4B6

Codon-optimized cDNA encoding the 4B6 variable heavy chain and variable light chain connected by a GGS(GGGGS)_3_ linker, were cloned into a derivative pCDNA3.1 vector in frame with an N-terminal human IgG1 signal sequence and a C-terminal thrombin-cleavable double StrepTagll affinity tag. A total of 120 µg of this plasmid was added to 8 × 10^7^ CHO-S cells and was electroporated. scFv 4B6 expression and purification were performed as described above for Fabs. Purified scFv was dialyzed into TBS. Synthetic genes for scFv 3H4 and 3B4 constructs were designed with the light and heavy chain variable domains connected by a GGS(GGGGS)_3_ linker and flanking BgIII and NheI restriction sites. The gene was codon optimized for *Drosophila melanogaster* and ordered from Integrated DNA Technologies. The gene was cloned into a pMT_puro_BiP vector via restriction digest in frame with an N-terminal BiP secretion signal and a C-terminal thrombin cleavable double StrepII affinity tag in the vector. pMT-puro_BiP vectors containing scFv 3H4 and scFv 3B4 were used to make stably transfected Schneider 2 (S2) cells as described previously (30). Expression and purification were performed as described previously (30).

### Expression and purification of recombinant neonatal Fc receptor

Codon-optimized cDNA encoding the ectodomain of the FCGRT gene (UniProt: P55899, Met1-Ser297) or the β-2-Microglobulin gene (UniProt: P61769, Met1-Met119) were cloned separately into a derivative pCDNA3.1 vector (35). The FCGRT construct also contained a C-terminal thrombin-cleavable double StrepTagll affinity tag. A total of 40 µg of FCGRT plasmid and 80 µg of β-2-Microglobulin plasmid were added to 8 × 10^7^ CHO-S cells and electroporated. CHO cell expression was performed as described above. The supernatant was loaded onto a StrepTrap XT affinity column (Cytiva), washed with Strep Wash Buffer, and eluted with Elution Buffer (Strep Wash containing 50 mM biotin). Purified FcRn was dialyzed into TBS.

### Binding assays of HAstV in the presence of neutralizing antibodies

Serial 1:5 dilutions of the ascitic fluids for 3B4, 3H4, or 4B6 (27), were pre-incubated, respectively, with infectious HAstV1 or HAstV2 particles purified as previously described (25), at a multiplicity of infection (MOI) or 30 (ffu/mL) for 1 h at room temperature. Caco-2 cell monolayers grown in 48-well plates were washed once with phosphate-buffered saline (PBS) pH 7.4, and then blocking solution (1% BSA in PBS) was added for 45 min at room temperature, followed by a 15 min incubation on ice. Cells were washed once with ice-cold PBS and incubated with the virus-antibody complex for 1 h on ice. MAb 2D9, which neutralizes HAstV8, was used as a negative control. The unbound virus was washed three times with cold PBS, and the total RNA was extracted with TRIzol Reagent (Invitrogen) according to the manufacturer’s instructions. Viral RNA or cellular 18S RNA was reverse transcribed using MMLV reverse transcriptase (Invitrogen). Quantitative reverse transcription PCR (RT-qPCR) was performed with the premixed reagent Real Q Plus Master Mix Green (Amplicon), and the PCR was carried out in an ABI Prism 7500 Detection System (Applied Biosystems). The primers used to detect HAstV1 RNA were Fwd 5′ - ATGAATTATTTTGATACTGAAGAAAATTACTTGGAA - 3′ and Rev 5′ - CTGAAGTACTTTGGTACCTATTTCTTAAGAAAG - 3′. For detection of HAstV2 RNA were Fwd 5′ - ATGAATTATTTTGATACTGAAGAAAGTTATTTGGAA - 3′ and Rev 5′ - CTGAAGTACTGTGGTACCTATTTCTTAAGAAAG - 3′. For normalization, 18S ribosomal cellular RNA was amplified and quantified using forward primer 5′ - CGAAAGCATTTGCCAAGAAT - 3′ and reverse primer 5′ - GCATCGTTTATGGTCGGAAC - 3′.

### Assay to determine if the neutralizing antibodies detach HAstV particles bound to cells

Confluent Caco-2 cell monolayers in 48-well plates were blocked with 1% BSA in PBS for 45 min at room temperature followed by a 15 min incubation on ice. Purified HAstV-1 or HAstV-2 particles were added at an MOI of 30 (ffu/mL) and then incubated for 1 h on ice to allow the binding of the virus to the cell surface while preventing virus internalization, which only occurs at 37°C. The unbound virus was subsequently removed by washing three times with cold PBS. Serial 1:5 dilutions of the indicated ascitic fluids of either 3B4 or 3H4 for HAstV1 or 4B6 for HAstV2 were added to the cells and then incubated for 1 h on ice. After this incubation, the antibody and unbound virus were removed with cold PBS, and RNA extraction and RT-qPCR quantification were performed as described above. MAb 2D9, which neutralizes HAstV8, was used as a negative control. Previous reports have used this virus detachment protocol, maintaining the cells on ice for rotavirus (36) and astrovirus studies (30), as well as for attachment studies on calicivirus (37) and rotavirus (38).

### X-ray crystallography structure determination of HAstV2 spike/scFv 4B6 complex

Thrombin digestion was used to remove the Histidine-tag from the HAstV2 spike and to remove the StrepII tag from scFv 4B6 (10 U thrombin/mg of protein incubated at 4°C on a rotating plane overnight). Digestion of affinity tags from scFv 4B6 and HAstV2 spike was confirmed by SDS-PAGE where no visually detectable undigested product was observed. HAstV2 spike was incubated with 2× molar excess scFv 4B6 per spike monomer and the resulting complex was purified by size-exclusion chromatography on a Superdex 75 10/300 Gl column. Fractions corresponding to the HAstV2 spike/scFv 4B6 complex were determined by peak comparison with gel filtration standards and SDS-PAGE analysis. HAstV2 spike/scFv 4B6 complex was concentrated to 5 mg/mL in TBS pH 8.5. HAstV2-spike/scFv 4B6 protein crystals were formed in 2 µL drops containing a 1:1 ratio of protein solution to well solution consisting of 0.1 M Tris-HCl pH 8.5, and 0.74 M sodium citrate pH 5.5, using hanging drop vapor diffusion at 22°C. A single crystal was transferred into a cryoprotectant solution consisting of well solution and 18% glycerol and was then flash-frozen into liquid nitrogen. The Advanced Photon Source synchrotron beamline 23-ID-D was used to collect a diffraction data set with wavelength 1.0332 Å at cryogenic temperatures. The data set was processed and scaled using DIALS (ccp4i2) (39) with a resolution cutoff of 2.67 Å based on CC_1/2_ and I/σl statistics. A trimmed model of HAstV2 spike (PDB: 3QSQ) and a trimmed model of scFv 4B6 generated by SWISS-model using tremelimumab Fab as a template (PDB: 5GGU) was used for molecular replacement with Phaser. The structure was then manually modeled using Coot (40) and refined in Phenix (41).

### Single-particle CryoEM structure determination of HAstV1 spike/Fab 3B4/Fab 3H4 complex

Thrombin digestion was used to remove affinity tags from the HAstV1 spike, Fab 3H4, and Fab 3B4 as described above. HAstV1 spike was complexed with 2× molar excess Fab 3B4 and the resulting complex was purified by SEC on Superdex 200 10/300 Gl column. Fractions corresponding to the HAstV1 spike/Fab 3B4 complex were determined by peak comparison with molecular weight standards and SDS-PAGE analysis. The resulting complex was then mixed with 1.5× molar excess Fab 3H4 and purified by SEC on a Superdex 200 10/300 Gl column. Fractions corresponding to the full HAstV1 spike/Fab 3B4/Fab 3H4 complex were determined by peak comparison with gel filtration standards and SDS-PAGE analysis. HAstV1 spike/Fab 3H4/Fab 3B4 complex was pooled and concentrated to 0.86 mg/mL in 10 mM Tris pH 7.0 and 150 mM NaCl. A 3 µL of protein complex was mixed with 0.5 µL of 25 µM lauryl maltose neopentyl glycol detergent to remove orientation bias and was then deposited onto glow discharged UltrAuFoil R.12/1.3 gold grids 400 mesh, blotted using a ThermoFisher Scientific (TFS) Vitrobot Mark IV at 4°C and 100% humidity, and then plunge frozen into liquid ethane. Grids were screened at UCSC’s Biomolecular CryoEM facility using a TFS Glacios 200 kV microscope coupled to a Gatan K2 Summit direct detector. The top-selected grids were then sent to the Pacific Northwest Center for Cryo-EM (PNCC #160263) for data collection on a TFS Krios G3i microscope coupled to a Gatan K3 Biocontinuum Gif.

7,235 movies containing 60 frames each were collected using a pixel size of 0.415 Å/pixel in super-resolution mode (105,000×) and an electron dose of 32.26 e/A (2). Movies were preprocessed (motion correction and CTF estimation) in CryoSPARC v4.3.2 (42). Initial particle identification was performed using an unbiased blob picker, resulting in 4,132,753 particles, further extracted in a box size of 686 pixels. After multiple rounds of 2D classification, 55 top-selected classes containing 214,273 particles underwent the *ab initio* reconstruction.

Three selected volumes were generated and then 3D-classified and further refined. The best-representing 3D class was used to create 2D references for a round of template picking. A total of 2,718,470 particles were extracted with a box size of 686 pixels and then underwent a similar previously established workflow. The top 72 classes containing 262,500 particles were used in a new *ab initio* reconstruction. The two generated classes were 3D-classified, where one class resulted in an overall gold-standard resolution (FSC_0.143_) of 5.70 Å and containing 138,147 particles. This volume was selected and underwent non-uniform refinement, and further non-uniform refinement using a mask encompassing the entire particle, resulting in a 3D reconstructed volume at 3.74 Å. Unused particles were added from the previous 2D classification, and all particles received local CTF refinement, resulting in a volume of 3.43 Å and 163,237 particles after non-uniform refinement. Additional rounds of local CTF refinement were performed and a mask in which the constant domains of the Fab were removed was used to align particles in local refinement, in order to improve the tridimensional alignment and local resolution of the epitope regions, resulting in the final reconstructed map at 3.33 Å overall resolution. The sharpened map (B factor −112 Å^2^) was opened in ChimeraX (version 1.5.0) and starting models of the HAstV1 spike (PDB: 5EWO) and AlphaFold 3 models of Fabs 3H4 and 3B4 were fitted into the volume. Since the Fab constant domain volume density was poor, the constant domains were removed from the models. The initial model representing the complex was opened in Coot (version 0.9.1), underwent several rounds of manual refinement and global real-space refinement and was validated using Phenix and MolProbity.

### Biolayer interferometry K_D_ determination of neutralizing antibodies 3B4, 3H4, and 4B6

Biolayer interferometry assays to determine Fab binding affinity dissociation constants (K_D_) were performed on an Octet RED384 instrument using Octet Kinetics Buffer (PBS pH 7.4 + 0.1% BSA + 0.02% Tween 20) for Fabs 3H4 and 3B4, or Octet Kinetics Buffer + biocytin (PBS pH 7.4 + 0.1% BSA + 0.02% Tween 20 + 50 µM biocytin) for Fab 4B6. For assays with Fabs 3H4 and 3B4, pre-equilibrated Anti-Penta-His (HIS1K) biosensor tips were dipped into Octet Kinetics Buffer for 60 s for an initial baseline reading, dipped into 0.5 µg/mL histidine-tagged HAstV1 spike diluted in Octet Kinetics Buffer for 180 s to load the sensor tip, and dipped into Octet Kinetics Buffer for 60 s for a second baseline reading. Biosensors were then dipped into 4-point serial dilutions of Fab in Octet Kinetics Buffer, consisting of 2.5, 5, 10, and 20 nM for Fab 3H4, and 20, 40, 80, and 160 nM for Fab 3B4. This association step was run for 180 s, and then biosensors were dipped into Octet Kinetics Buffer to measure dissociation for a total of 600 s. Assays to determine the K_D_ of Fab 4B6 to HAstV2 spike were run the same way except the HAstV2 spike was loaded to biosensors, the serial dilution series consisted of 25, 50, 100, and 200 nM 4B6 Fab, and the association and dissociation steps were 60 s each. These shorter association and dissociation steps were chosen due to the lower affinity of the Fab 4B6. Kinetics data were processed the same way for all Fabs in the Data Analysis HT software. The baseline step was used to align traces and apply the inter-step correction. A reference sample well containing only a spike-loaded biosensor dipped into no analyte (Fab) was subtracted. Savitzky-Golay filtering was used on the traces. For curve fitting, a 1:1 model was globally applied to the dilution series, and the fit was evaluated based on R^2^ and χ^2^ values and visual inspection. Average K_D_ values are reported as the average of the three replicates.

### Biolayer interferometry competition assay of Fabs versus FcRn for HAstV spike

Using an Octet RED384, pre-equilibrated Streptavidin (SA) biosensor tips were dipped into the Octet Kinetics Buffer for 60 s for an initial baseline reading. For Fabs 3H4 and 3B4, 0.5 µg/mL of biotinylated HAstV1 spike was loaded onto SA biosensors tips for 300 s and dipped into Octet Kinetics Buffer for 30 s for a baseline reading. Biosensors were then dipped into either 150 nM Fab 3H4 or 250 nM Fab 3B4 in Octet Kinetics Buffer for 600 s to ensure saturation of all spike binding sites, dipped into Octet Kinetics Buffer for 30 s as a baseline reading, and then dipped into 2 µM FcRn in Octet Kinetics Buffer for 300 s. For Fab 4B6, the assay was performed with the same methods, but 0.5 µg/mL biotinylated HAstV2 spike was used during the antigen loading step, Octet Kinetics buffer + biocytin was used for all assay steps after antigen loading, 250 nM Fab 4B6 was using during the antibody association step, and due to its lower affinity, 250 nM of Fab 4B6 was also included in the baseline step after antibody association and in the FcRn sample to maintain saturation. All assays contained additional control in which the primary Fab was dipped into the same concentration of self Fab instead of FcRn to ensure saturation occurred, and also a control in which FcRn was bound to the HAstV spike in the absence of any Fab in the first association. An additional control was run using 2 µM of unrelated protein, GFP, instead of FcRn, to show specificity. All assays were performed in triplicate. A matrix representing competition was generated in the Data Analysis HT software in the Epitope Binning module using the shift between the last 10% average of the signal from the second association and the last 10% average of the signal from the primary association step. The signal from the control in which primary Fab was dipped into self Fab for the second association was subtracted in the matrix row (the shift values from all samples containing the respective Fab), such that full competition is represented by “0.” The signal from the control sample in which FcRn was associated with HAstV spike with no Fab in the primary association was used to normalize in the matrix column (the shift values from samples with FcRn binding in the secondary association step) such that maximum FcRn binding with no competition represents “1.” This normalization is done separately for Fab 4B6 vs Fab 3H4 and 3B4 assays given the different spike serotypes to which FcRn is associated but is displayed in the same table. Values were converted into percentages. Significance was determined using a one-tailed independent *t*-test using the raw data values before normalization: *P < 0.05; **P < 0.01; ***P < 0.001; ****P < 0.0001. Fabs were considered to compete with FcRn if FcRn binding in the presence of Fab was reduced by 50% or more (a value of 0.5 or lower). The values shown are an average of triplicate assays.

### scFv 3B4, scFv 3H4, and 4B6 neutralization assays

The indicated concentration of antibody or scFv was preincubated with HAstV1 (for scFv 3H4, scFv 3B4, and mAb 3B4) or HAstV2 (for scFv 4B6) at an MOI of 0.02 (ffu/mL), for 1 h at room temperature. The virus-antibody mixture was then added to confluent Caco-2 cell monolayers grown in 96-well plates and incubated for 1 h at 37°C. After this time, the cells were washed three times with minimum essential medium without serum, and the infection was left to proceed for 18 h at 37°C. Infected cells were detected by an immunoperoxidase focus-forming assay, as described previously (27).

## RESULTS

### HAstV-neutralizing antibodies 3B4, 3H4, and 4B6 bind with high affinity to the HAstV spike

We previously generated a panel of IgG1 monoclonal antibodies (mAbs), three of which were found to neutralize either HAstV1 (mAbs 3B4 and 3H4) or HAstV2 (mAb 4B6) in Caco-2 cells, the gold standard cell line used for HAstV propagation and infectivity studies (43). Here, we generated recombinant antigen-binding fragments (Fabs) of these HAstV-neutralizing antibodies to remove the avidity effects of a full bivalent mAb given the homodimeric nature of their target, the HAstV capsid spike domain. To determine binding affinities, biosensors loaded with HAstV spike were dipped into serial dilutions of Fabs. All three Fabs bind the corresponding HAstV spike with high affinities, with dissociation constants (K_D_s) in the mid-low nanomolar range (Table 1; Fig. S1). These results indicate that mouse immunization with recombinant HAstV spikes is able to induce high-affinity HAstV-neutralizing antibodies in cell culture (27). Interestingly, Fab 4B6 has the lowest affinity of the three antibodies, yet has the most potent neutralizing activity (27). This observation may indicate that other factors besides affinity, such as the location of the antibody binding site or avidity, may influence HAstV neutralization, or this result could be related to serotype difference.

**TABLE 1:** Antibodies 3B4, 3H4, and 4B6 bind HAstV spike with high affinity

Antibody to spike	Average K_D_ ± σ (nM)	χ²	R²
Fab 3H4 – HAstV1 spike	0.490 ± 0.002	<0.2339	>0.9991
Fab 3B4 – HAstV1 spike	11.8 ± 0.5	<0.7474	>0.9909
Fab 4B6 – HAstV2 spike	161 ± 2	<0.0675	>0.9943

### HAstV-neutralizing antibodies 3B4, 3H4, and 4B6 block attachment of HAstV to Caco-2 cells

**Fig 1:**
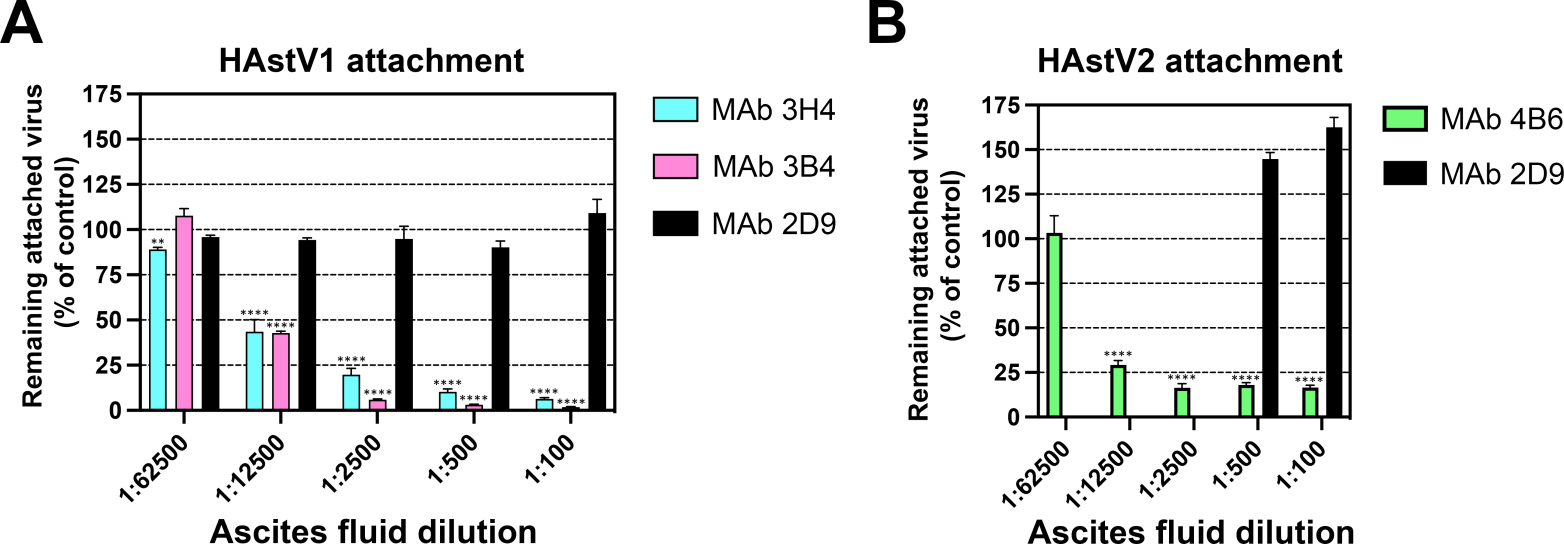
Monoclonal antibodies to HAstV1 and HAstV2 block attachment of the virus to Caco-2 cells. Different dilutions of ascitic fluids of mAbs 3B4 or 3H4 to HAstV1 (**A**) or mAb 4B6 to HAstV2 (**B**) were preincubated with the purified corresponding virus for 1 h at 37°C and then the virus-antibody complexes were added to Caco-2 cell monolayers for 1 h on ice to allow the virus to attach to the cell surface. After removing the unbound virus and washing the cells, the attached virus was determined by RT-qPCR as described in Materials and Methods. MAb 2D9, specific to serotype HAstV8, was used as a negative control. Experiments were performed on ice to prevent virus endocytosis. The assay was performed in biological quintuplicates and carried out in duplicate. The data are expressed as percentages of the virus attached without antibodies and represent the mean ± SEM. Significance was determined using a one-way analysis of variance (ANOVA). *P < 0.05; **P < 0.01; ***P < 0.001; ****P < 0.0001. In both panels, the significance of the various 3H4, 3B4, and 4B6 antibody dilutions is referred to as the 1:100 2D9 ascitic fluid.

**Fig 2:**
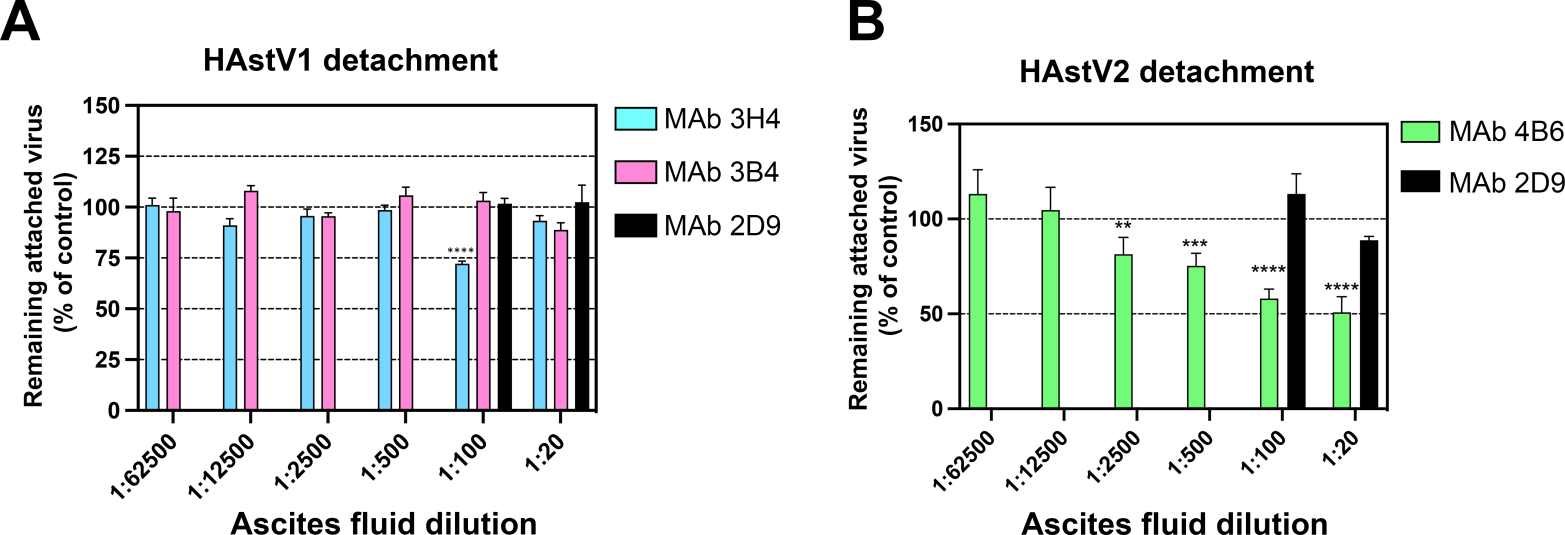
Monoclonal antibody detachment of HAstV previously bound to the surface of Caco-2 cells. (**A**) HAstV1 or (**B**) HAstV2 was attached to cells on ice to prevent virus endocytosis. Subsequently, ascitic fluid of (**A**) mAb 3B4 or 3H4 or (**B**) mAb 4B6 was added to cells and incubated for 1 h on ice. After washing the cells, the remaining attached virus was determined by RT-qPCR as described in Materials and Methods. MAb 2D9, specific to serotype HAstV8, was used as a negative control. In both panels, only the two highest concentrations of Mab 2D9 were used as a control. The assay was performed in biological sextuplicates and carried out in duplicate. The data are expressed as percentages of the virus that remained attached in the absence of antibodies and represent the mean ± SEM. Significance was determined using ANOVA. *P < 0.05; **P < 0.01; ***P < 0.001; ****P < 0.0001. In both panels, the significance of the various 3H4, 3B4, and 4B6 antibody dilutions is referred to as the 1:100 2D9 ascitic fluid.

To further investigate the mechanism of antibody neutralization, we tested whether mAbs 3B4, 3H4, or 4B6 could block the attachment of HAstV to Caco-2 cells, and whether these antibodies could detach the virus which was already bound to cells. Caco-2 monolayers were incubated with HAstV-antibody complexes or HAstV alone. The unbound virus was washed away and the bound virus was quantified using RT-qPCR. We found that all three antibodies were able to block virus attachment to cells in a dose-dependent manner compared to a negative control antibody (Fig. 1). Interestingly, only 4B6 was able to partially detach pre-bound virus at the highest concentration of ascitic fluid tested (Fig. 2).

### HAstV-neutralizing antibodies 3B4, 3H4, and 4B6 compete with FcRn binding to HAstV spike

With the recent discovery of FcRn as a critical receptor for HAstV infection, we investigated whether the HAstV-neutralizing antibodies 3B4, 3H4, and 4B6 could compete with FcRn’s ability to bind to the HAstV spike (28, 29). Either HAstV1 or HAstV2 spikes were loaded onto biosensors and then dipped into saturating levels of Fab 3H4, 3B4, or 4B6. The biosensors were then dipped into FcRn and these binding shifts were compared to the binding shifts of FcRn to spike-loaded biosensors in the absence of Fab. From this assay, we determined that Fabs 3H4 and 4B6 fully block FcRn binding (Table 2) to 4% and 6%, respectively, suggesting that these Fabs either directly or sterically block FcRn’s ability to bind the spike protein. Fab 3B4 does not appear to fully block FcRn binding but reduces FcRn binding to 40% of the control. Given that 3B4 is still efficient at neutralizing HAstV1, Fab 3B4 may have an alternative mechanism of neutralizing HAstV, such as blocking the interaction of another putative receptor, or the full-length mAb may be necessary for full steric hindrance of the FcRn interaction with HAstV1 spike. These data suggest that one mechanism of antibody neutralization may be by blocking the FcRn interaction with the HAstV spike.

**TABLE 2:** Antibodies 3B4, 3H4, and 4B6 compete with FcRn receptor to HAstV spike

	FcRn binding ± σ (%)	Significance
Fab 3B4	40 ± 9	** (*P* < 0.01)
Fab 3H4	4 ± 2	** (*P* < 0.01)
Fab 4B6	6.0 ± 0.3	** (*P* < 0.01)
No Fab	100	

### HAstV2-neutralizing antibody 4B6 binds to a distinct epitope on the upper loops of the HAstV2 spike

**Fig 3:**
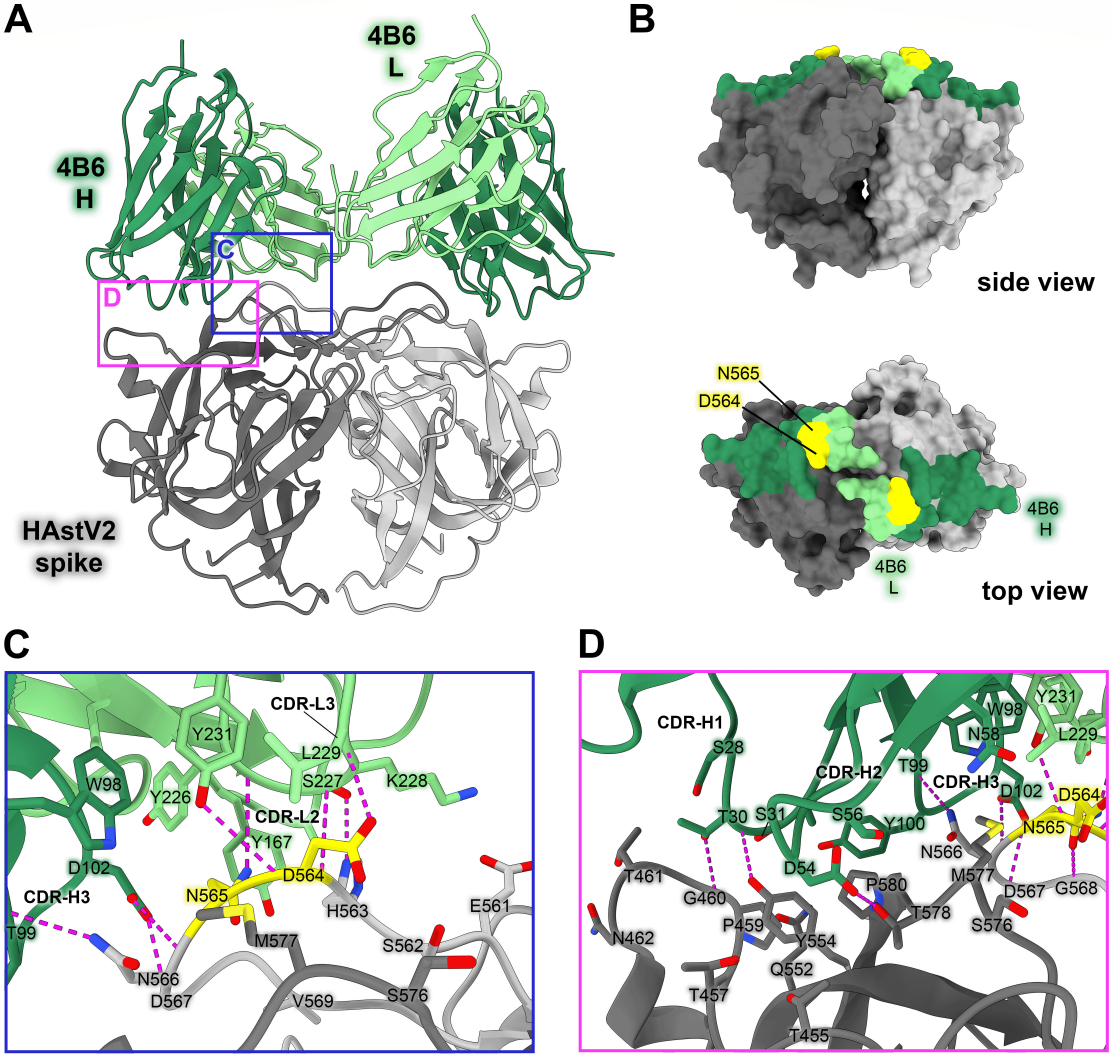
Neutralizing antibody 4B6 binds to a unique epitope on the top of the HAstV2 spike. (**A**) Crystal structure of scFv 4B6 bound to the HAstV2 spike homodimer solved to 2.67 Å resolution and displayed as a ribbon model. The spike is colored gray and scFv 4B6 is colored green, with the heavy chain colored dark green and the light chain colored light green. Pink and blue panels show the locations of the focused views shown in panels C and D. (**B**) Surface model of the HAstV2 spike with residues involved in the 4B6 epitope colored in dark green for heavy chain interactions or light green for light chain interactions. The yellow residues indicate previously identified escape mutation locations to antibody 4B6 (27). (**C**) Focused view on the light chain interaction, with 4B6 light chain colored light green. Side chains involved in hydrogen bonding are shown, with hydrogen bonds colored magenta. 4B6 light chain predominantly interacts with spike loop 3. (**D**) Focused view on the heavy chain interaction, with 4B6 heavy chain colored dark green. 4B6 heavy chain predominantly interacts with beta sheets 8 and 11, and the tip of loop 3 on the HAstV spike.

**Fig 4:**
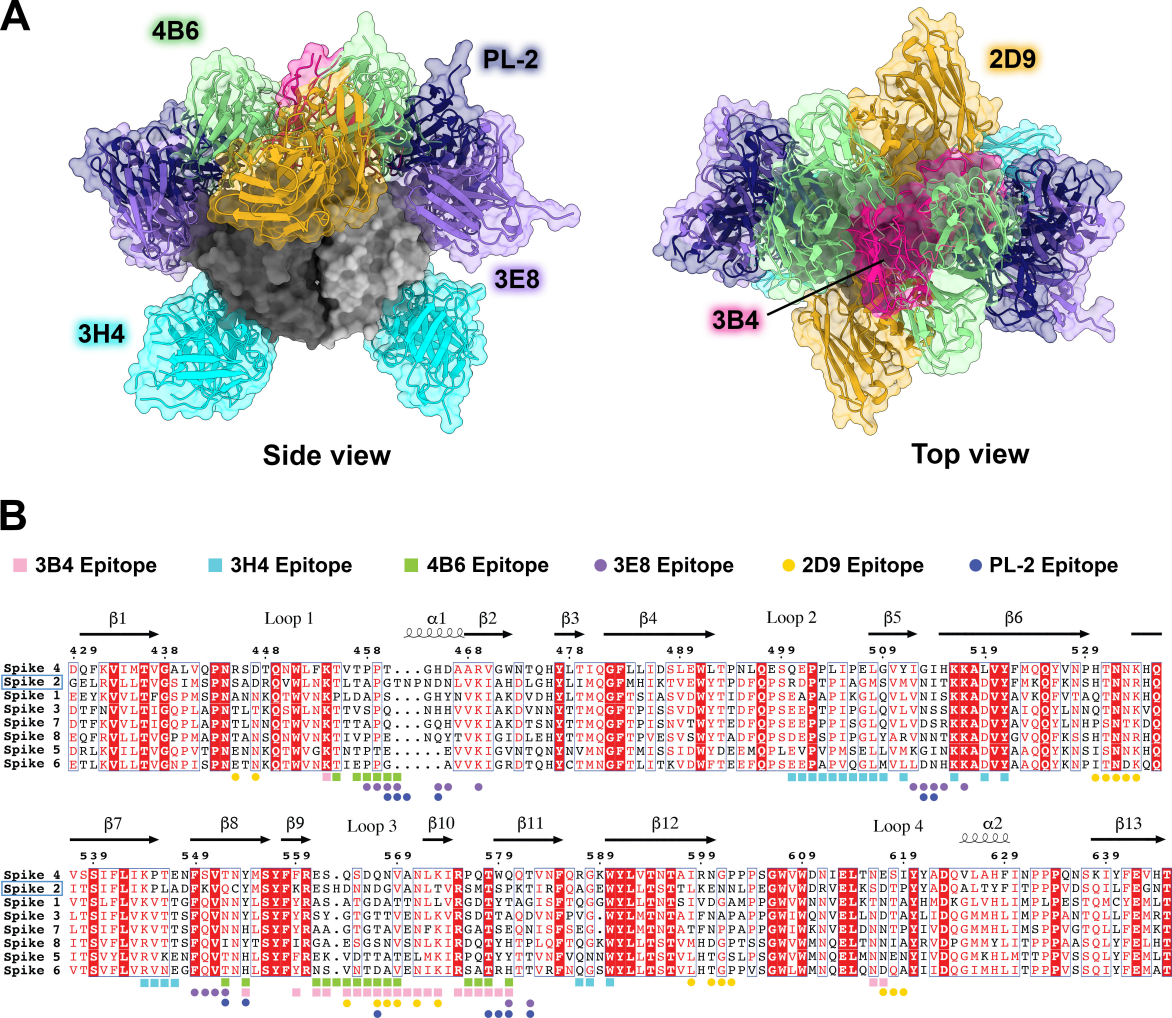
Comparison of all known HAstV-neutralizing antibody epitopes, showing that most target the upper variable region of HAstV spike. (**A**) Alignment of all existing HAstV neutralizing antibody structures 4B6, 3B4, 3H4, 3E8, 2D9, and PL-2, mapped onto HAstV1 spike. (**B**) Spike protein sequences of the eight classical HAstV serotypes aligned using EMBL-MUSCLE, with residues colored according to conservation. The following sequences were used for the alignment: HAstV1, GenBank #AAC34717.1; HAstV2, GenBank #KY964327.1; HAstV3, UniProt #Q9WFZ0.1; HAstV4, UniProt #Q3ZN05.1; HAstV5, UniProt #Q4TWH7.1; HAstV6, UniProt #Q67815.1; HAstV7, UniProt #Q96818.2; HAstV8, UniProt #Q9IFX1.2. The residue numbering shown above corresponds with HAstV2. Residues highlighted in red are strictly conserved, residues with red text are semi-conserved, and residues in black text have little to no conservation. Spike residues interacting with the antibodies characterized in this paper, 3H4, 3B4, and 4B6, are indicated with colored squares, and epitope residues for antibodies that were previously characterized, 2D9, 3E8, and PL-2, are indicated as colored circles.

Currently, the epitopes for only three HAstV-neutralizing monoclonal antibodies have been structurally defined, which revealed two immunogenic sites on the spike (30, 31). Subsequently, we sought to characterize three additional neutralizing antibodies, 3B4, 3H4, and 4B6, to determine if other immunogenic sites on the HAstV spike exist. Previous escape mutation studies identified two adjacent amino acid changes in the HAstV2 spike, D564E and N565D, which allowed HAstV2 to overcome the neutralizing activity of antibody 4B6 (27). However, the epitope of mAb 4B6 has not been structurally defined. To visualize where neutralizing antibody 4B6 binds to the HAstV spike, we solved the crystal structure of the recombinant single-chain variable fragment (scFv) 4B6 in complex with the HAstV2 spike to 2.67 Å resolution (Fig. 3; Table 3). This structure revealed that 4B6 binds to a novel 694 Å^2^ quaternary epitope at the top of the spike. Each chain of 4B6 interacts predominantly with the long loop 3 from the opposing protomer, with some residues in the CDR-H3 loop of the heavy chain interacting with both protomers (Fig. 3C and D). All 3 CDRs in the 4B6 heavy chain interact with the spike, but in the light chain, only CDR-L1 and CDR-L3 interact. Antibody 4B6 forms a network of 8 hydrogen bonds with spike residues 563–567 at the very tip of loop 3, which interact with both light chain CDR L3 residues Y226-Y231 and heavy chain CDR H3 residues D102 and T99 (Fig. 3C). This hydrogen bond network consists of a mix of side-chain and backbone interactions for both the antibody and spike. This data correlates with the two residues D564 and N565 on loop 3 that were previously identified as locations for escape mutations to antibody 4B6—the mutation of these two residues would disrupt at least two hydrogen bond interactions, which may explain how these escape mutations disrupt 4B6 neutralization of HAstV (Fig. 3B) (27). The HAstV spike loop 3, which 4B6 primarily targets, is highly variable across strains of HAstV (Fig. 4), which may indicate that this location is particularly immunogenic and frequently targeted by antibodies, creating selective pressure for the virus to mutate this region.

**TABLE 3:** Crystallographic statistics for scFv 4B6/HAstV2 spike complex (PDB entry 9CN2)

Characteristic	Value
Data collection	
Space group	P 41 3 2
Cell dimensions	
a, b, c (Å)	160.35, 160.35, 160.35
α, β, γ (°)	90, 90, 90
Resolution (Å)	160.64–2.67 (2.72–2.67)
Rmerge	0.191 (3.291)
Rpim	0.022 (0.438)
I/σl	23.4 (1.0)
Completeness	100% (100%)
Multiplicity	74.5 (55.5)
CC_1/2_	0.999 (0.755)
Refinement	
Resolution (Å)	40.09–2.67 (2.77–2.67)
No. reflections for refinement	20,582
No. reflections for R_free_	1,997 (197)
R_work_/R_free_	0.223/0.265
No. atoms	3,496
Protein	3,493
Ligand/ion	0
Water	3
B-factors (Å^2^)	72.28
Protein	72.29
Ligand/ion	N/A
Water	60.18
Protein residues	444
RMSD	
Bond lengths	0.009
Bond angles	1.11
Ramachandran statistics	
Favored (%)	96.58
Allowed (%)	3.42
Outliers (%)	0

### HAstV1-neutralizing antibody 3H4 binds to a novel epitope near the base of the spike, and HAstV1-neutralizing antibody 3B4 binds the top dimer interface in a unique asymmetric way

**Fig 5:**
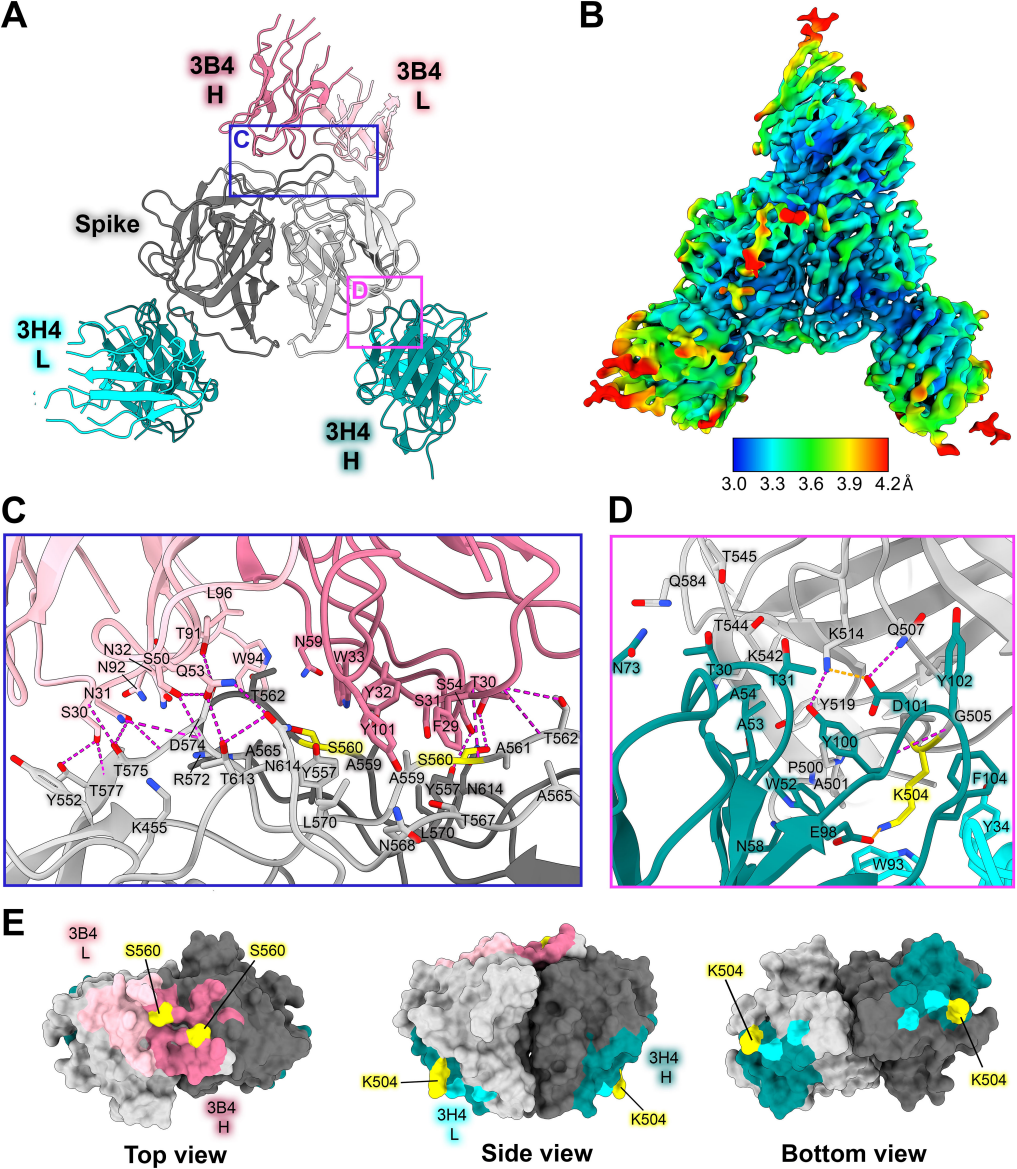
Neutralizing antibody 3H4 binds to a unique epitope at the base of the spike, and neutralizing antibody 3B4 has a unique top epitope in which a single antibody binds the spike dimer interface. (**A**) Single-particle cryoEM reconstructed map solved to FSC_0.143_ 3.33 Å of neutralizing Fab 3H4 and Fab 3B4 bound simultaneously to the HAstV1 spike, displayed as a ribbon model with 3H4 colored cyan and 3B4 colored pink. The heavy and light chains are colored in dark and light shades, respectively. Pink and blue panels show the locations of the focused views shown in panels C and D. (**B**) Local resolution estimation of the cryoEM structure of HAstV1 spike bound to 3H4 Fab and 3B4 Fab, with contour level at 0.043 in ChimeraX. (**C**) Focused view of the 3B4 epitope, with the light chain colored light pink, and the heavy chain colored dark pink, with hydrogen bond interactions colored magenta. Serine 560, which was previously identified as a residue that overcomes the neutralization activity of 3B4 when mutated to proline, is highlighted in yellow. (**D**) Focused view of the 3H4 epitope, with the light chain colored light cyan, and the heavy chain colored dark teal. Hydrogen bond interactions are colored magenta and salt bridges are colored in orange. Lysine 504, which was previously identified as a residue that overcomes the neutralization activity of 3H4 when mutated to glutamic acid, is highlighted in yellow. (**E**) Surface view of the HAstV1 spike with antibody interacting residues colored according to antibody chain. Residues interacting with both chains are colored according to the predominant interaction. Residues that confer resistance to the respective antibody when mutated are colored in yellow.

Previous escape mutation studies revealed a single point mutation K504E (for 3H4) or S560P (for 3B4) in the HAstV1 spike that allowed the virus to escape the neutralizing effects of antibody 3H4 or 3B4 (27). To define the full epitopes of antibodies 3H4 and 3B4, we solved the structure of both Fabs 3H4 and 3B4 in complex with the HAstV1 spike to 3.33 Å resolution using single-particle cryoEM (Fig. 5A and B; Fig. S2; Table 4). This structure reveals two novel epitopes, with a single Fab 3B4 bound to the top of the spike dimer interface, and two Fab 3H4 bound to the bottom sides of the spike dimer (Fig. 5A and B). Antibody 3B4 spans a 1,039 Å^2^ quaternary epitope across the top dimer interface, with more of the epitope located on one protomer than the other (Fig. 5A and E). Based on the structure, as well as the retention volume of the complex in solution on a size-exclusion chromatography column, only one Fab 3B4 can bind the spike homodimer at a time, which represents the first antibody of its kind to be discovered for HAstV, as all other previously characterized antibodies can bind symmetrically with one antibody binding site per protomer (30, 31). Antibody 3B4 targets the majority of loop 3 on one monomer closer to the base of the loop, and the side of loop 3 on the other monomer. Although 3B4 targets a similar structural region on the HAstV1 spike as that of 4B6 on the HAstV2 spike, it interacts with unique residues focused more on the center of the dimer interface, while 4B6 is targeted more outward toward the tips of loop 3. All six of the 3B4 CDR loops interact with the spike, forming a hydrogen bond network primarily between spike residues G573-T577, and 3B4 residues S30-N32 on CDR-L1 (Fig. 5C). Q53 and S50 from CDR-L2 also contribute several hydrogen bonds, with Q53 making two hydrogen bonds with T613 and N614. On the heavy chain, the majority of the hydrogen bonds are contributed by CDR-H1 residues T28 and T30, which target T562 and S560 on the spike. Although a single point mutation of S560P in the spike sequence confers resistance to antibody 3B4 neutralization (27), this mutation changes two distinct sites of interaction with the heavy chain of 3B4, given the close locations of each S560 to each other on the dimer interface (Fig. 5C and E). This could suggest that single point mutations offer higher resistance to dimer interface antibodies in comparison to antibodies that bind both protomers. Despite both 4B6 and 3B4 targeting loop 3, 3B4 targets residues that are more conserved (Fig. 4B).

**TABLE 4:** Statistics for cryoEM structure of Fab 3B4/Fab 3H4/HAstV1 spike complex

Data collection information	PNCC #160258—Krios-3
Nominal magnification	105,000×
Voltage (kV)	300
Electron dose (e^-^/Å^2^)	32.26
Physical pixel size (super-res) (Å)	0.415
Movies amount	7,235
Defocus average and range (µm)	−1.5 (−2.5 to −0.5)
Frames	60

Single-particle reconstruction information	EMDB 45427
Initial particles picked	4,132,753
Particles in the final map	163,237
Initial model used	PDB 5EWO, *ab initio*
Symmetry imposed	C1
Map overall resolution—FSC_0.143_ (Å)	3.33
Map resolution range (Å)	3.0–4.2
Map sharpening	
B-factor (Å^2^)	−112

Built model information	PDB 9CBN
Chains	11
Atoms (non-H)	10,967
Protein residues	1,040
Water	0
Ligands	
BMA	2
NAG:	5
Bonds (RMSD)	
Length (Å, # >4σ)	0.020 (2)
Angles (°, # >4σ)	1.536 (91)
Mean B-factor (Å^2^)	
Protein	0.78/102.01/40.47
Nucleotide	N/A
Ligand	30.00/56.36/50.22
Water	N/A
MolProbity score	2.72
Clashscore	31.17
Ramachandran plot	
Favored (%)	92.24
Allowed (%)	7.76
Outliers (%)	0
Rama-Z (Ramachandran plot Z-score, RMSD)	
Whole (*N* = 3,625)	−2.05 (0.26)
Helix (*N* = 1,443)	−5.16 (0.42)
Sheet (*N* = 472)	−1.26 (0.26)
Loop (*N* = 1,710)	−1.36 (0.25)
Rotamer outliers (%)	2.22
Cβ outliers (%)	1.77
Peptide plane (%)	
*Cis* proline/general	7.3/0.0
Twisted proline/general	0.0/0.0
CaBLAM outliers (%)	3.49
Occupancy	
Mean	1
occ = 1 (%)	99.26
0 < occ < 1 (%)	0.74
occ > 1 (%)	0
Model vs data	
Mean CC for ligands	0.64
CC (peaks)	0.64
CC (volume)	0.75
CC (box)	0.7
CC (mask)	0.78

Fab 3H4 binds to a novel 676 Å^2^ epitope near the base of the spike which is distinct from any other known HAstV-neutralizing antibody epitopes, as all previously solved spike-antibody structures target the top or upper sides of the spike dimer (Fig. 5A and B, Fig. 4A). The 3H4 epitope interaction is facilitated almost entirely by the heavy chain alone, with only W93 from CDR L1 and Y34 CDR L3 from the light chain making any contact with the spike (Fig. 5D and E). Antibody 3H4 mostly targets the upper portion of the spike loop 2 with all three heavy chain CDR loops. Two salt bridges formed between K514 on spike and D101 on CDR-H3, and K504 with E98 on CDR-H3 (Fig. 5D). These lysine residues also form hydrogen bonds and cation-pi interactions with Fab 3H4. Notably, the salt bridge interaction by K504 appears critical to the ability of 3H4 to bind to spike as the mutation of K504 to a negatively-charged glutamic acid disrupts 3H4 neutralization of HAstV1 (27). Antibody 3H4 also targets a region of much higher conservation than that of the other neutralizing antibodies (30, 31), with over 70% of the interacting spike residues being semi-conserved or higher amongst the 8 HAstV serotypes shown in Fig. 4B. Despite the majority of residues being conserved, K504, which is critical to 3H4 neutralization, is highly variable among serotypes, which likely accounts for the 3H4 serotype specificity (27).

### 3B4, 3H4, and 4B6 scFv neutralize HAstV

**Fig 6:**
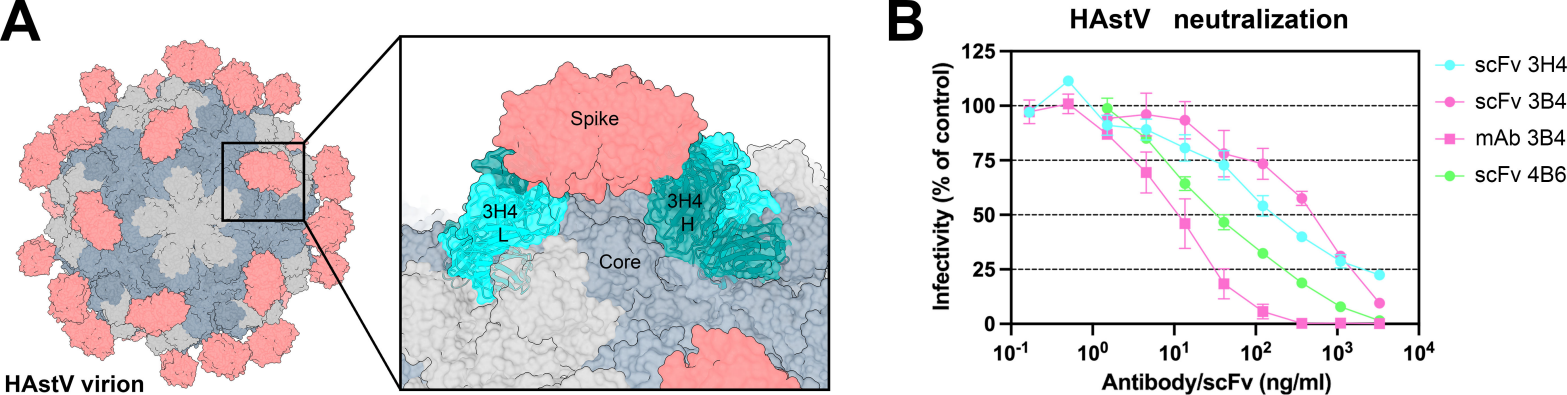
Steric hindrance of antibody 3H4 constant domains may play a role in its ability to neutralize HAstV1. (**A**) Graphic depicting the full virion capsid, with the core domains colored in gray and the spike domains colored in salmon. The panel shows a focused view of how Fab 3H4 would clash with the HAstV capsid core, using the cryoEM reconstruction of the 3H4 variable domain aligned with an AlphaFold 3 model of the constant domain. (**B**) Neutralization activity of scFv 3H4, scFv 3B4, and mAb 3B4 against HAstV1, or scFv 4B6 against HAstV2. HAstV was preincubated with the corresponding scFv or mAb at the indicated concentrations. The infectivity of the virus was determined as described in Materials and Methods. The infectivity assay was performed in biological triplicates and carried out in duplicate. The data are expressed as % infectivity of control and represent the mean ± SEM.

Antibody 3H4 reveals a particularly interesting epitope location, as the full-length antibody would likely clash with the icosahedral core of the HAstV capsid (Fig. 6A), suggesting that this antibody may contort the spike dimer in some way. Since 3H4 binds so distantly from other structurally determined neutralizing antibody epitopes and yet is still shown to block FcRn receptor binding (4%), we hypothesized that 3H4 may neutralize HAstV by steric hindrance with its constant regions and contortion of the spike, rather than the direct blocking of an important functional site on the spike. We tested whether scFv 3H4, scFv 3B4, and scFv 4B6 which lack antibody constant domains, could still neutralize HAstV1 (scFv 3H4 and scFv 3B4) or HAstV2 (scFv 4B6). HAstV1 was preincubated with serial dilutions of scFv 3H4, scFv 3B4, or mAb 3B4 as a control, or HAstV2 with scFv 4B6, and was incubated on a Caco-2 cell monolayer, and viral infection was measured by an immunoperoxidase focus-forming assay. We found that both the scFv 3H4, 3B4, and 4B6 are still able to neutralize HAstV and do so to a similar degree, but are not as effective at neutralization as full-length mAb (Fig. 6B), indicating that steric hindrance and/or avidity have a role in the ability of these antibodies to neutralize the virus. 4B6 appears to be the most effective at neutralization of HAstV2 despite its lower affinity compared to 3B4 and 3H4, indicating that antibody affinity may not always be directly correlated with neutralization ability, and other factors such as epitope location may also play a role.

### AlphaFold 3 prediction accuracy

**Fig 7:**
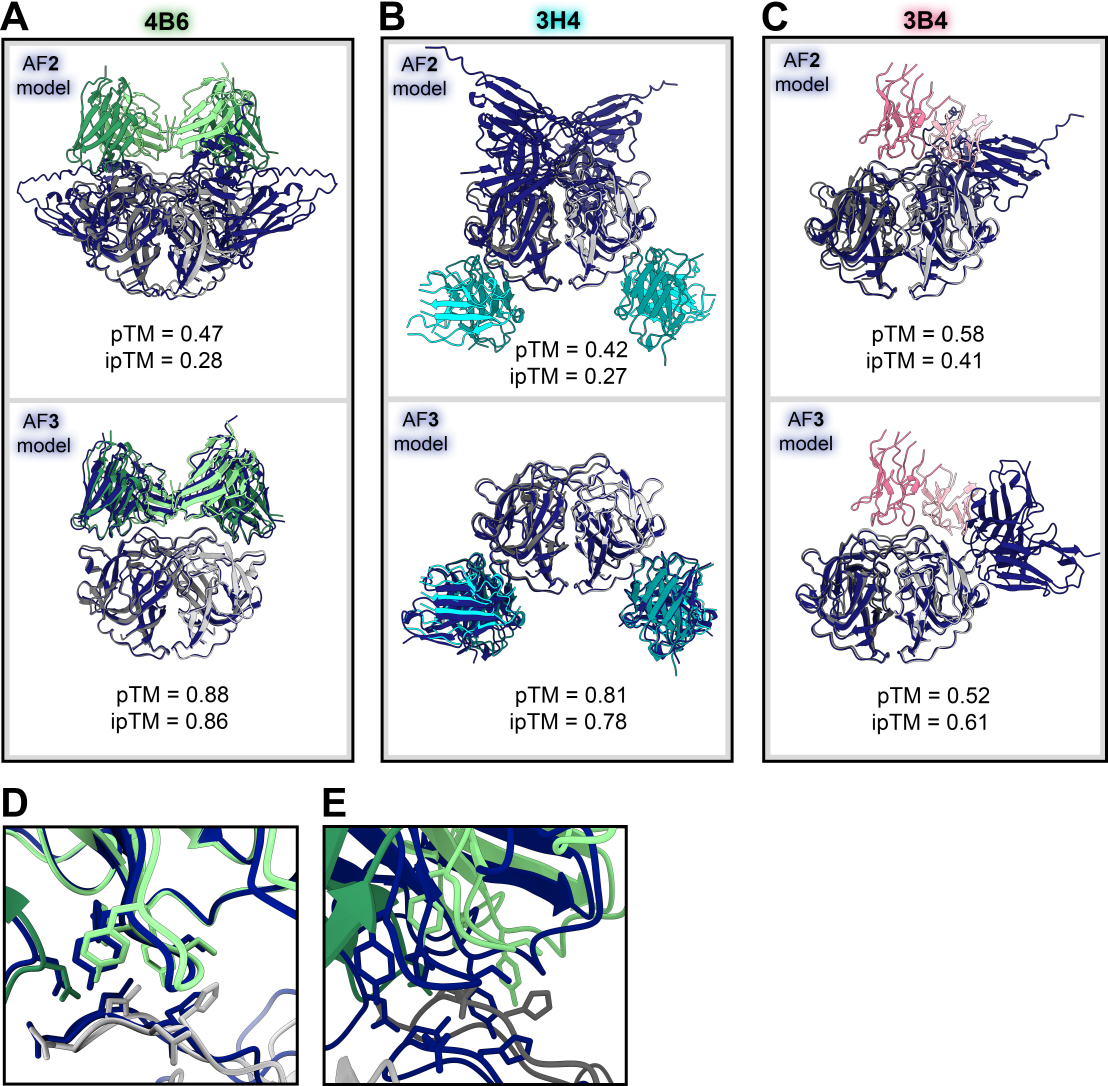
AlphaFold 3-predicted models of scFv 4B6 bound to HAstV2 spike and Fab 3H4 bound to HAstV1 are highly accurate. (**A**) Crystal structure of antibody scFv 4B6 bound to HAstV2 spike (in green and gray) aligned in ChimeraX with the AlphaFold 2 predicted model (in navy blue, above) or AlphaFold 3 predicted model (in navy blue, below). (**B**) CryoEM structure of the variable domain of Fab 3H4 bound to HAstV1 spike (in cyan and gray) aligned in ChimeraX with the AlphaFold 2 predicted model (in navy blue, above) or AlphaFold 3 predicted model (in navy blue, below). (**C**) CryoEM structure of the variable domain of Fab 3B4 bound to HAstV1 spike (in pink and gray) aligned in ChimeraX with the AlphaFold 2 predicted model (in navy blue, above) or AlphaFold 3 predicted model (in navy blue, below). (**D**) Focused view of the crystal structure light chain CDR-L3 loop interacting with the tip of HAstV2 spike loop 3 aligned with the AlphaFold 3 predicted model. (**E**) Focused view of the opposing spike protomer at the same location as in panel D.

As predictive protein structural software advances, we sought to assess how accurately the recent release of AlphaFold 3 (AF3) could predict antibody-antigen interactions (44). We compared the crystal structure of scFv 4B6 and HAstV2 spike with that of its AlphaFold prediction and found that not only was the antibody placed correctly, but even the side chain interactions were highly accurate (Fig. 7A). However, on a macroscopic scale, the dimer interfaces appear to be slightly misaligned, causing the other protomer alignment and subsequent interacting residues to be slightly misaligned (Fig. 7D and E), though the local side chain orientations still appear to be highly accurate. The observation that AlphaFold has particular difficulty with correct prediction of the dimer interface interaction has been seen with other astrovirus spike predictions (45). AF3 was confident in its prediction, with a pTM of 0.88 and ipTM of 0.86. The overall accuracy of the AF3 model is quite high, with a TM-score of 0.97 (TM value of 1 = identical match) when the AF3 model is aligned to the crystal structure. This is a substantial improvement from the AlphaFold 2 (AF2) prediction, which did not place scFv 4B6 in the correct general placement, let alone correct side chain orientations (Fig. 7A). We additionally compared the AF3 model of Fab 3H4 and Fab 3B4 with our solved cryoEM structure. The AF3 model of Fab 3H4 bound to the HAstV1 spike was highly accurate (Fig. 7B), with a TM-score of 0.99 when aligned to the cryoEM structure with Fab 3B4 removed. Despite the higher TM score, AF3 reported slightly lower confidence scores, with ipTM = 0.78, and pTM = 0.81. The AF3 predicted model for 3H4 additionally showed dramatic improvement from the AlphaFold 2 (AF2) prediction, which did not place Fab 3H4 in the correct general placement. In the case of Fab 3B4, AF3 could not successfully find the correct general placement and consistently placed 3B4 Fab on the side of the spike dimer (Fig. 7C), even when we tried alternative searches for one or two Fabs or scFvs. Because the overall interface alignment appears to be slightly off in these AF3 models, this may explain why AF3 could not predict the epitope of Fab 3B4 correctly, which targets the dimer interface. AF3 was less confident in its predicted model of one Fab 3B4 bound to HAstV1 spike, with ipTM = 0.52 and pTM = 0.61, but were still above the 0.5 threshold suggesting that the structure could be correct despite being an incorrect placement. However, the decrease in these scores for 3B4 compared to 3H4 and 4B6 does suggest some ability to determine whether the predicted structure is correct. From these assessments, AlphaFold 3 appears to have a dramatic increase in accuracy compared to previous versions which consistently failed to predict antibody interactions at all, even though some challenging antibodies that target interfaces may still be more difficult.

## DISCUSSION

Here, we mapped three new epitopes on the HAstV spike that induce neutralizing antibodies and found that 4B6 and 3B4 target the top of the spike in ways that are unique from previously characterized antibodies. Additionally, we found that 3H4 targets the base of the spike, representing a unique epitope that is distant from previously characterized antibodies and targets mostly conserved residues. With these additional structures, we found that the majority of neutralizing antibodies target the upper side or top variable loop regions of the spike (Fig. 4). These regions reside around conserved areas of the HAstV spike, termed the P-site and S-site, which were proposed as potential host protein interacting (46). However, few of the antibody residues directly target these conserved sites. Although the direct receptor or host protein interaction locations on the spike are currently not known, it is possible that these neutralizing antibodies sterically hinder receptor binding to more conserved residues, rather than overlapping with receptor binding site(s) directly, given that the majority of neutralizing antibodies target highly variable loop residues on the top regions of the spike, indicating less functional importance for these residues. Therefore, these loops may serve more as an immunogenic target for antibodies that can be more easily mutated without changing important functions of the spike. This is similarly seen in other enteric viruses such as norovirus, in which the majority of antigenic sites map to outer loop regions (47). For norovirus, it is suggested that mutations in loops more easily result in structural changes that do not affect the stability of the P domain but still affect the overall surface landscape and contribute to serotype specificity (47). This may also be the case for astrovirus, as spike loops show higher variation and structural conformations compared to other regions of the spike, and in one study, a structural change in loop 1 on HAstV2, prevented neutralizing antibody PL-2 from binding to the spike (32).

3H4 represents an entirely new antigenic site near the base of the spike, which has less accessibility compared to the top exposed portion of the spike where the majority of antibodies target. This low epitope may have been favored more by recombinant spike immunization than what would have been induced by the whole virus where the capsid core domain limits access. This suggests the possibility of using recombinant spike vaccinations for enhancing the induction of less accessible antigenic sites that may be more conserved on the spike, similar to how some recombinant influenza vaccine antigen candidates better elicit immune-subdominant hemagglutinin stem targeting antibodies (48, 49). Given that 3H4 targets mostly conserved residues and has high affinity, it may have more potential as a monoclonal antibody therapy for HAstV1 than other antibodies that bind more variable residues. However, 3H4 is vulnerable to mutations at residue K504, which additionally is not conserved between HAstV serotypes.

All three antibodies block the attachment of HAstV to cells, but only 4B6 can partially detach the virus which has already been bound, despite 4B6 having the lowest affinity of the three antibodies discussed here. We previously reported that mAbs 2D9 (neutralization titer 1:5 × 10^6^) and 3E8 (neutralization titer 1:1 × 10^6^) to HAstV8 also detach the previously bound virus (27). Thus, it seems that the ability of the various mAbs to detach prebound virus particles correlates with the neutralization titer, considering the neutralization titers of mAb 4B6 to HAstV2 (1:800,000) as compared to mAbs 3H4 (1:20,000) and 3B4 (1:16,000) to HAstV1 (27). Similar findings have been reported for rotavirus (36) and rhinovirus (50). In this regard, neutralization titer appears to correlate with detachment ability more than affinity, as both 4B6 (K_D_: ~161 nM) and 3E8 (K_D_: ~40 nM), which can partially detach bound virus in a dose-dependent manner, have lower affinities than 3B4 (K_D_: ~12 nM) and 3H4 (K_D_: ~0.5 nM), which cannot (27). It is also possible that lower affinity antibodies with higher avidity could play a role in the nAb’s ability to neutralize. Although 4B6 is only able to partially detach the virus, this is seen in the other two antibodies, 2D9 and 3E8, which can also detach the virus but also only to partial levels at the lowest dilution tested (1:100) (27). Again, this detachment appears to correlate with neutralization titer, as 2D9 which has the highest neutralization titer, was also most effective at detaching. 3E8, with the next highest neutralization titer, is the next most effective at detachment, and 4B6 with a lower neutralization titer than 2D9 or 3E8, is also less effective at detaching virus than 2D9 or 3E8 at the same dilution (27). It is interesting, however, that the only two antibodies in our panel that cannot detach the virus both target HAstV1. It is unknown whether this is a coincidence resulting from a relatively small panel of antibodies or whether there is a mechanism unique to serotype 1 that makes it more difficult to detach from cells, such as an additional unknown attachment factor, given that both 3H4 and 3B4 are higher affinity than 4B6 or 3E8 and also block or partially block FcRn attachment. Differences in receptor usage within the same family of viruses are not unheard of, as some strains of norovirus use HGBAs as attachment factors while others do not (51). A larger panel of neutralizing antibodies toward HAstV1 would be helpful to distinguish whether this inability of 3H4 and 3B4 to detach is specific to serotype 1, or unique to these two antibodies in particular. A combination of a high concentration of neutralizing antibodies with enough affinity to compete for the interaction between the virus spike and cellular receptor, especially if antibodies are directed specifically at the cellular attachment site on the virus, might be expected to elute bound virions.

Understanding which regions of the astrovirus spike protein induce neutralizing antibodies that block receptor binding could help inform its use as a vaccine antigen, as the correlation between receptor-blocking neutralizing antibodies *in vitro* and protection from infection or severe disease *in vivo* has been seen in other gastroenteritic viruses such as norovirus (52). We find that all three antibodies discussed here, 3H4, 4B6, and 3B4, block the binding of FcRn to the spike, although 3B4 only appears to partially block FcRn binding to around 40%. From the structure, it can be seen that 3B4 leans more to one side of the spike dimer than the other. It is possible that this asymmetric nature of the 3B4 binding antibody could explain how only partial blocking of FcRn binding occurs if FcRn were to bind both sides of the spike homodimer and 3B4 was capable of only blocking one side. It is interesting that all three antibodies block FcRn binding given their different locations on the spike, which leads to our hypothesis that the blocking ability of these antibodies may be more related to steric hindrance and less related to where the antibodies bind directly. This does seem to be the case given that the scFvs of 3H4 and 3B4 neutralize less effectively than full-length mAb, however, they are still able to neutralize virus at higher concentrations, indicating that there may still be some overlap with the receptor-binding site(s), or that some steric hindrance still occurs with the variable region.

Overall, these studies further our structural and mechanistic understanding of neutralizing antibody epitopes on the HAstV capsid surface, supporting the rational design of vaccines targeting HAstV spikes to prevent childhood viral diarrhea by HAstV.

## Data Availability

Coordinates and structure factors for the HAstV2 spike/scFv 4B6 complex structure were deposited in the Protein Data Bank under accession code 9CN2. For the HAstV1 spike/Fab 3B4/Fab 3H4 complex structure, the final reconstructed map was deposited in the Electron Microscopy Data Bank under accession code EMD-45427, and the final model was deposited into the Protein Data Bank under accession code 9CBN.

## SUPPLEMENTAL MATERIAL

Supplemental material jvi.01619-24-s0001.pdf Figures S1 to S3.

ASM does not own the copyrights to Supplemental Material that may be linked to, or accessed through, an article. The authors have granted ASM a non-exclusive, world-wide license to publish the Supplemental Material files. Please contact the corresponding author directly for reuse.

## References

[1] Olortegui MP, Rouhani S, Yori PP, Salas MS, Trigoso DR, Mondal D, Bodhidatta L, Platts-Mills J, Samie A, Kabir F, et al.. 2018. Astrovirus infection and diarrhea in 8 countries. Pediatrics 141:e20171326. doi:10.1542/peds.2017-1326 29259078 PMC9923568

[2] Ghosh S, Kumar M, Santiana M, Mishra A, Zhang M, Labayo H, Chibly AM, Nakamura H, Tanaka T, Henderson W, Lewis E, Voss O, Su Y, Belkaid Y, Chiorini JA, Hoffman MP, Altan-Bonnet N. 2022. Enteric viruses replicate in salivary glands and infect through saliva. Nature New Biol 607:345–350. doi:10.1038/s41586-022-04895-8 PMC9243862 35768512

[3] Kurtz JB, Lee TW, Craig JW, Reed SE. 1979. Astrovirus infection in volunteers. J Med Virol 3:221–230. doi:10.1002/jmv.1890030308 113503

[4] Daniel-Wayman S, Fahle G, Palmore T, Green KY, Prevots DRN. 2018. Norovirus, astrovirus, and sapovirus among immunocompromised patients at a tertiary care research hospital. Diagn Microbiol Infect Dis 92:143–146. doi:10.1016/j.diagmicrobio.2018.05.017 29934072 PMC11036324

[5] Osborne CM, Montano AC, Robinson CC, Schultz-Cherry S, Dominguez SR. 2015. Viral gastroenteritis in children in Colorado 2006-2009. J Med Virol 87:931–939. doi:10.1002/jmv.24022 25776578 PMC7166904

[6] Wunderli W, Meerbach A, Güngör T, Berger C, Greiner O, Caduff R, Trkola A, Bossart W, Gerlach D, Schibler M, Cordey S, McKee TA, Van Belle S, Kaiser L, Tapparel C. 2011. Astrovirus infection in hospitalized infants with severe combined immunodeficiency after allogeneic hematopoietic stem cell transplantation. PLoS ONE 6:e27483. doi:10.1371/journal.pone.0027483 22096580 PMC3214048

[7] Black RE, Perin J, Yeung D, Rajeev T, Miller J, Elwood SE, Platts-Mills JA. 2024. Estimated global and regional causes of deaths from diarrhoea in children younger than 5 years during 2000-21: a systematic review and Bayesian multinomial analysis. Lancet Glob Health 12:e919–e928. doi:10.1016/S2214-109X(24)00078-0 38648812 PMC11099298

[8] Acosta AM, Chavez CB, Flores JT, Olotegui MP, Pinedo SR, Trigoso DR, Vasquez AO, Ahmed I, Alam D, Ali A, et al.. 2014. The MAL-ED study: a multinational and multidisciplinary approach to understand the relationship between enteric pathogens, malnutrition, gut physiology, physical growth, cognitive development, and immune responses in infants and children up to 2 years of age in resource-poor environments. Clin Infect Dis 59:S193–S206. doi:10.1093/cid/ciu653 25305287

[9] Lee TW, Kurtz JB. 1984. Human astrovirus serotypes. Lancet 324. doi:10.1016/S0140-6736(84)92101-9 6150404

[10] Jeong HS, Jeong A, Cheon D-S. 2012. Epidemiology of astrovirus infection in children. Korean J Pediatr 55:77–82. doi:10.3345/kjp.2012.55.3.77 22474461 PMC3315622

[11] Tao Z, Lin X, Liu Y, Ji F, Wang S, Xiong P, Zhang L, Xu Q, Xu A, Cui N. 2022. Detection of multiple human astroviruses in sewage by next generation sequencing. Water Res 218:118523. doi:10.1016/j.watres.2022.118523 35525029

[12] Vu D-L, Cordey S, Brito F, Kaiser L. 2016. Novel human astroviruses: novel human diseases? J Clin Virol 82:56–63. doi:10.1016/j.jcv.2016.07.004 27434149

[13] Koukou G, Niendorf S, Hornei B, Schlump J-U, Jenke AC, Jacobsen S. 2019. Human astrovirus infection associated with encephalitis in an immunocompetent child: a case report. J Med Case Reports 13:341. doi:10.1186/s13256-019-2302-6 PMC6874811 31757225

[14] Lu R-G, Li S-S, Hu B, Li H-Y, Tian H, Liu W-Q, Yan X-J, Liu H, Bai X. 2022. The first evidence of shaking mink syndrome-astrovirus associated encephalitis in farmed minks, China. Transbound Emerg Dis 69:3979–3984. doi:10.1111/tbed.14693 36057957

[15] Comeau D, Spinato MT, Ojkic D, Foster RA, Caswell JL. 2024. Bovine astrovirus and its role in lymphocytic encephalitis in cattle in Ontario, Canada, 1988-2019. J Vet Diagn Invest 36:447–456. doi:10.1177/10406387241237192 38500024 PMC11110778

[16] Wildi N, Seuberlich T. 2021. Neurotropic astroviruses in animals. Viruses 13:1201. doi:10.3390/v13071201 34201545 PMC8310007

[17] Meyer L, Delgado-Cunningham K, Lorig-Roach N, Ford J, DuBois RM. 2021. Human astrovirus 1-8 seroprevalence evaluation in a United States adult population. Viruses 13:979. doi:10.3390/v13060979 34070419 PMC8229645

[18] Koopmans MPG, Bijen MHL, Monroe SS, Vinjé J. 1998. Age-stratified seroprevalence of neutralizing antibodies to astrovirus types 1 to 7 in humans in the Netherlands. Clin Diagn Lab Immunol 5:33–37. doi:10.1128/CDLI.5.1.33-37.1998 9455876 PMC121387

[19] Farahmand M, Khales P, Salavatiha Z, Sabaei M, Hamidzade M, Aminpanah D, Tavakoli A. 2023. Worldwide prevalence and genotype distribution of human astrovirus in gastroenteritis patients: a systematic review and meta-analysis. Microb Pathog 181:106209. doi:10.1016/j.micpath.2023.106209 37385570

[20] Björkholm M, Celsing F, Runarsson G, Waldenström J. 1995. Successful intravenous immunoglobulin therapy for severe and persistent astrovirus gastroenteritis after fludarabine treatment in a patient with Waldenström’s macroglobulinemia. Int J Hematol 62:117–120. doi:10.1016/0925-5710(95)00396-a 8590772

[21] Banos-Lara MR, Méndez E. 2010. Role of individual caspases induced by astrovirus on the processing of its structural protein and its release from the cell through a non-lytic mechanism. Virology (Auckl) 401:322–332. doi:10.1016/j.virol.2010.02.028 20347468

[22] Méndez E, Fernández-Luna T, López S, Méndez-Toss M, Arias CF. 2002. Proteolytic processing of a serotype 8 human astrovirus ORF2 polyprotein. J Virol 76:7996–8002. doi:10.1128/jvi.76.16.7996-8002.2002 12134004 PMC155114

[23] Bass DM, Qiu S. 2000. Proteolytic processing of the astrovirus capsid. J Virol 74:1810–1814. doi:10.1128/jvi.74.4.1810-1814.2000 10644354 PMC111659

[24] Dryden KA, Tihova M, Nowotny N, Matsui SM, Mendez E, Yeager M. 2012. Immature and mature human astrovirus: structure, conformational changes, and similarities to hepatitis E virus. J Mol Biol 422:650–658. doi:10.1016/j.jmb.2012.06.029 22743104 PMC7127750

[25] Aguilar-Hernández N, López S, Arias CF. 2018. Minimal capsid composition of infectious human astrovirus. Virology (Auckl) 521:58–61. doi:10.1016/j.virol.2018.05.021 29883775

[26] Bass DM, Upadhyayula U. 1997. Characterization of human serotype 1 astrovirus-neutralizing epitopes. J Virol 71:8666–8671. doi:10.1128/JVI.71.11.8666-8671.1997 9343224 PMC192330

[27] Espinosa R, López T, Bogdanoff WA, Espinoza MA, López S, DuBois RM, Arias CF. 2019. Isolation of neutralizing monoclonal antibodies to human astrovirus and characterization of virus variants that escape neutralization. J Virol 93:e01465-18. doi:10.1128/JVI.01465-18 30355681 PMC6321937

[28] Ingle H, Molleston JM, Hall PD, Bui D, Wang L, Bhatt KD, Foster L, Antia A, Ding S, Lee S, Fremont DH, Baldridge MT. 2024. The neonatal Fc receptor is a cellular receptor for human astrovirus. Nat Microbiol 9:3321–3331. doi:10.1038/s41564-024-01855-y 39578577 PMC11970254

[29] Haga K, Tokui T, Miyamoto K, Takai-Todaka R, Kudo S, Ishikawa A, Ishiyama R, Kato A, Yokoyama M, Katayama K, Nakanishi A. 2024. Neonatal Fc receptor is a functional receptor for classical human astrovirus. Genes Cells 29:983–1001. doi:10.1111/gtc.13160 39266307 PMC11555631

[30] Ricemeyer L, Aguilar-Hernández N, López T, Espinosa R, Lanning S, Mukherjee S, Cuellar C, López S, Arias CF, DuBois RM. 2022. Structures of two human astrovirus capsid/neutralizing antibody complexes reveal distinct epitopes and inhibition of virus attachment to cells. J Virol 96:e0141521. doi:10.1128/JVI.01415-21 34613806 PMC8754201

[31] Bogdanoff WA, Campos J, Perez EI, Yin L, Alexander DL, DuBois RM. 2017. Structure of a human astrovirus capsid-antibody complex and mechanistic insights into virus neutralization. J Virol 91:e01859–16, doi:10.1128/JVI.01859-16 27807234 PMC5215351

[32] Bogdanoff WA, Perez EI, López T, Arias CF, DuBois RM. 2018. Structural basis for escape of human astrovirus from antibody neutralization: broad implications for rational vaccine design. J Virol 92:e01546-17. doi:10.1128/JVI.01546-17 PMC5730766 29070688

[33] York RL, Yousefi PA, Bogdanoff W, Haile S, Tripathi S, DuBois RMS. 2016. Structural, mechanistic, and antigenic characterization of the human astrovirus capsid. J Virol 90:2254–2263. doi:10.1128/JVI.02666-15 PMC4810704 26656707

[34] Meyer L, López T, Espinosa R, Arias CF, Vollmers C, DuBois RM. 2019. A simplified workflow for monoclonal antibody sequencing. PLoS One 14:e0218717. doi:10.1371/journal.pone.0218717 31233538 PMC6590890

[35] Moritz B, Becker PB, Göpfert U. 2015. CMV promoter mutants with a reduced propensity to productivity loss in CHO cells. Sci Rep 5:16952. doi:10.1038/srep16952 26581326 PMC4652263

[36] Ruggeri FM, Greenberg HB. 1991. Antibodies to the trypsin cleavage peptide VP8 neutralize rotavirus by inhibiting binding of virions to target cells in culture. J Virol 65:2211–2219. doi:10.1128/JVI.65.5.2211-2219.1991 1850007 PMC240568

[37] White LJ, Ball JM, Hardy ME, Tanaka TN, Kitamoto N, Estes MK. 1996. Attachment and entry of recombinant Norwalk virus capsids to cultured human and animal cell lines. J Virol 70:6589–6597. doi:10.1128/JVI.70.10.6589-6597.1996 8794293 PMC190699

[38] Zárate S, Espinosa R, Romero P, Méndez E, Arias CF, López S. 2000. The VP5 domain of VP4 can mediate attachment of rotaviruses to cells. J Virol 74:593–599. doi:10.1128/jvi.74.2.593-599.2000 10623720 PMC111578

[39] Winter G, Beilsten-Edmands J, Devenish N, Gerstel M, Gildea RJ, McDonagh D, Pascal E, Waterman DG, Williams BH, Evans G. 2022. DIALS as a toolkit. Protein Sci 31:232–250. doi:10.1002/pro.4224 34747533 PMC8740827

[40] Emsley P, Lohkamp B, Scott WG, Cowtan K. 2010. Features and development of Coot. Acta Crystallogr D Biol Crystallogr 66:486–501. doi:10.1107/S0907444910007493 20383002 PMC2852313

[41] Liebschner D, Afonine PV, Baker ML, Bunkóczi G, Chen VB, Croll TI, Hintze B, Hung L-W, Jain S, McCoy AJ, Moriarty NW, Oeffner RD, Poon BK, Prisant MG, Read RJ, Richardson JS, Richardson DC, Sammito MD, Sobolev OV, Stockwell DH, Terwilliger TC, Urzhumtsev AG, Videau LL, Williams CJ, Adams PD. 2019. Macromolecular structure determination using X-rays, neutrons and electrons: recent developments in Phenix. Acta Cryst D Struct Biol 75:861–877. doi:10.1107/S2059798319011471 31588918 PMC6778852

[42] Punjani A, Rubinstein JL, Fleet DJ, Brubaker MA. 2017. cryoSPARC: algorithms for rapid unsupervised cryo-EM structure determination. Nat Methods 14:290–296. doi:10.1038/nmeth.4169 28165473

[43] De Benedictis P, Schultz-Cherry S, Burnham A, Cattoli G. 2011. Astrovirus infections in humans and animals - molecular biology, genetic diversity, and interspecies transmissions. Infect Genet Evol 11:1529–1544. doi:10.1016/j.meegid.2011.07.024 21843659 PMC7185765

[44] Abramson J, Adler J, Dunger J, Evans R, Green T, Pritzel A, Ronneberger O, Willmore L, Ballard AJ, Bambrick J, et al.. 2024. Accurate structure prediction of biomolecular interactions with AlphaFold 3. Nature New Biol 630:493–500. doi:10.1038/s41586-024-07487-w PMC11168924 38718835

[45] Wang Y, Guo X, Chang S, Zhao L, Li A, Liu X, Ma H, Li Y. 2024. Mamastrovirus spike protein: sequence and structural characterization as a basis for understanding cross-species transmission. Int J Biol Macromol 282:137366. doi:10.1016/j.ijbiomac.2024.137366 39537052

[46] Dong J, Dong L, Méndez E, Tao Y. 2011. Crystal structure of the human astrovirus capsid spike. Proc Natl Acad Sci U S A 108:12681–12686. doi:10.1073/pnas.1104834108 21768348 PMC3150915

[47] Shanker S, Czako R, Sankaran B, Atmar RL, Estes MK, Prasad BVV. 2014. Structural analysis of determinants of histo-blood group antigen binding specificity in genogroup I noroviruses. J Virol 88:6168–6180. doi:10.1128/JVI.00201-14 24648450 PMC4093872

[48] Krammer F, Palese P. 2013. Influenza virus hemagglutinin stalk-based antibodies and vaccines. Curr Opin Virol 3:521–530. doi:10.1016/j.coviro.2013.07.007 23978327 PMC3804342

[49] Yassine HM, Boyington JC, McTamney PM, Wei C-J, Kanekiyo M, Kong W-P, Gallagher JR, Wang L, Zhang Y, Joyce MG, Lingwood D, Moin SM, Andersen H, Okuno Y, Rao SS, Harris AK, Kwong PD, Mascola JR, Nabel GJ, Graham BS. 2015. Hemagglutinin-stem nanoparticles generate heterosubtypic influenza protection. Nat Med 21:1065–1070. doi:10.1038/nm.3927 26301691

[50] Colonno RJ, Callahan PL, Long WJ. 1986. Isolation of a monoclonal antibody that blocks attachment of the major group of human rhinoviruses. J Virol 57:7–12. doi:10.1128/JVI.57.1.7-12.1986 3001366 PMC252692

[51] Zhan X, Li Q, Tian P, Wang D. 2024. The attachment factors and attachment receptors of human noroviruses. Food Microbiol 123:104591. doi:10.1016/j.fm.2024.104591 39038896

[52] Reeck A, Kavanagh O, Estes MK, Opekun AR, Gilger MA, Graham DY, Atmar RL. 2010. Serological correlate of protection against norovirus-induced gastroenteritis. J Infect Dis 202:1212–1218. doi:10.1086/656364 20815703 PMC2945238

