# Supplementary material for: Discovery of three novel neutralizing antibody epitopes on the human astrovirus capsid spike and mechanistic insights into virus neutralization
Source: J Virol. 2025 Jan 23;99(2):e01619-24. doi: 10.1128/jvi.01619-24 (PMC11852706; doi:10.1128/jvi.01619-24)
Supplement: Supplemental material — Figures S1 to S3. [file jvi.01619-24-s0001.pdf]

Supplemental Figures

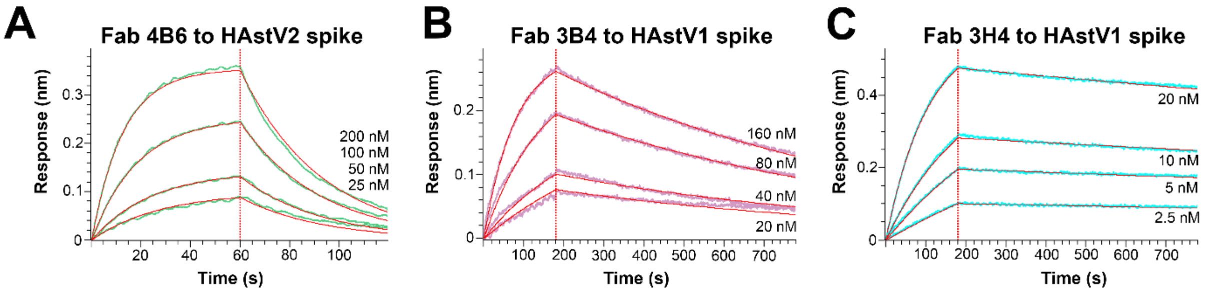

Supplemental Figure 1: Antibodies 3H4, 3B4 and 4B6 bind HAstV spike with high affinity. (A) Biolayer interferometry (BLI) traces of a representative Fab 4B6 dilution series (green) with global curve fits (red). (B) BLI traces of a representative Fab 3B4 dilution series (pink) with global curve fit (red). (C) BLI traces of a representative Fab 3H4 dilution series (cyan) with global curve fit (red).

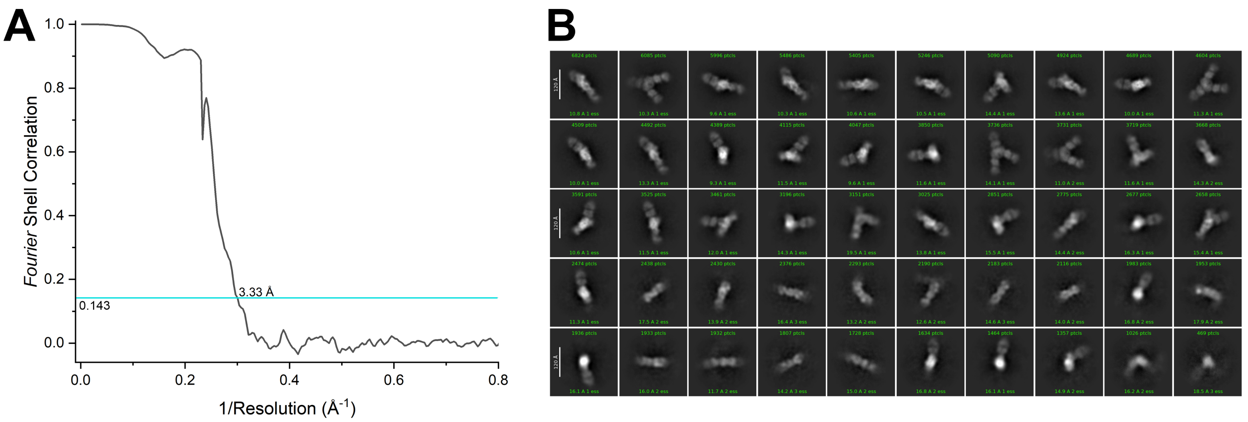

Supplemental Figure 2: Fab 3B4 and 3H4 bound to HAstV1 spike single-particle cryoEM reconstruction (A) $FSC_{0.143}$ with an overall resolution of 3.33 Å. (B) Representative 2D classes.

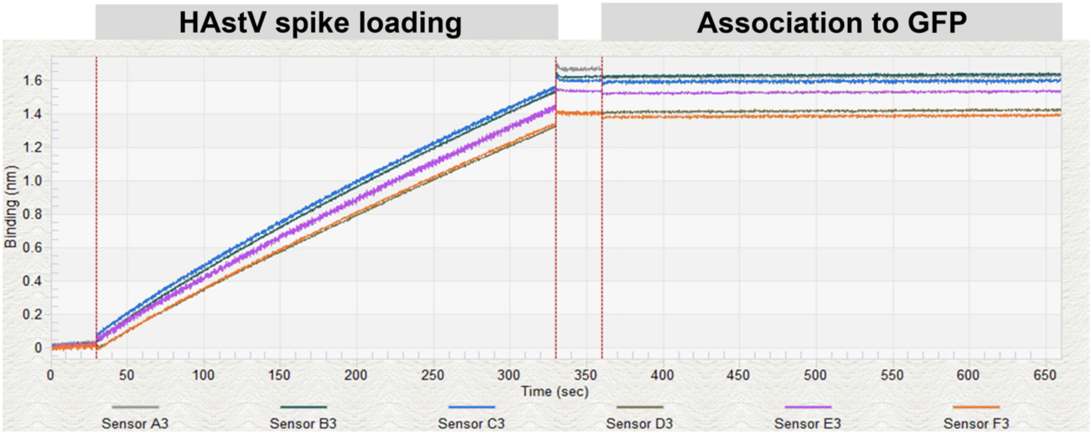

Supplemental Figure 3: Binding of FcRn to HAstV spike is specific and not observed with control protein GFP. Raw BLI traces of HAstV1 spike (sensors A3, B3, C3) or HAstV2 spike (sensors D3, E3, F3) coated biosensors dipped into unrelated control protein GFP at 2 µM.
